# The utility of airborne hyperspectral and satellite multispectral images in identifying Natura 2000 non-forest habitats for conservation purposes

**DOI:** 10.1038/s41598-023-31705-6

**Published:** 2023-03-20

**Authors:** Anna Jarocińska, Dominik Kopeć, Jan Niedzielko, Justyna Wylazłowska, Anna Halladin-Dąbrowska, Jakub Charyton, Agnieszka Piernik, Dariusz Kamiński

**Affiliations:** 1 Department of Geoinformatics, Cartography and Remote Sensing, Chair of Geomatics and Information Systems, Faculty of Geography and Regional Studies, University of Warsaw, Krakowskie Przedmieście 26/28, 00-927 Warszawa, Poland; 2 Department of Biogeography, Paleoecology and Nature Conservation, Faculty of Biology and Environmental Protection, University of Lodz, Banacha 1/3, 90-237 Łódź, Poland; 3 MGGP Aero Sp. z o.o., Kaczkowskiego 6, 33-100 Tarnów, Poland; 4 Department of Geobotany and Landscape Planning, Faculty of Biological and Veterinary Sciences, Nicolaus Copernicus University in Toruń, Lwowska 1, 87-100 Toruń, Poland

**Keywords:** Macroecology, Biodiversity, Image processing, Machine learning

## Abstract

Aerial hyperspectral and multispectral satellite data are the two most commonly used datasets to identify natural and semi-natural vegetation. However, there is no documented analysis based on data from several areas concerning the difference in the classification accuracy of non-forest Natura 2000 habitat with the use of aerial hyperspectral and satellite multispectral data. Also, there is no recommendation, on which habitat can be classified with sufficient accuracy using free multispectral images. This study aimed to analyse the difference in classification accuracy of Natura 2000 habitats representing: meadows, grasslands, heaths and mires between data with different spectral resolutions and the results utility for nature conservation compared to conventional maps. The analysis was conducted in five study areas in Poland. The classification was performed on multispectral Sentinel-2 (S2) and hyperspectral HySpex (HS) images using the Random Forest algorithm. Based on the results, it can be stated that the use of HS data resulted in higher classification accuracy, on average 0.14, than using S2 images, regardless of the area of the habitat. However, the difference in accuracy was not constant, varying by area and habitat characterisation. Greater differences in accuracy were observed for areas where habitats were characterised by high α-diversity or β-diversity. The HS and S2 data make it possible to create maps that provide a great deal of new knowledge about the distribution of Natura 2000 habitats, which is necessary for the management of protected areas. The obtained results indicate that by using S2 images it is possible to identify, at a satisfactory level, alluvial meadows and grassland. For heaths and mires, using HS data improved the results, but it is also possible to acquire general distribution of these classes, whereas HS images are obligatory for mapping salt, *Molinia* and lowland hay meadows.

## Introduction

Anthropogenic environmental changes cause an increasing number of habitats to be threatened with extinction every year^1^. In Europe, the protection of natural and semi-natural habitats was introduced throughout the European Union to establish a list of protected habitats^2^. In order to protect these habitats, Special Areas of Conservation (SACs) were designated, and in 2021 the EU and United Kingdom area covered 945,785 km^2,3^, which will further increase according to The EU Biodiversity Strategy for 2030^4^. At present, this can be considered one of the most important initiatives supporting biodiversity protection in the world^5^. Based on the Directive, each EU country is obliged to monitor the condition of its protected habitats. The traditional methods of habitat monitoring based on field mapping have numerous limitations, so intensive research is carried out on the development of new technologies for habitat monitoring, e.g., remote sensing (RS)^6,7^. RS techniques can be an alternative to the traditional method of acquiring information, and may also become a tool that will help solve one of the biggest problems on a national scale—the lack of information on the distribution of protected natural habitats outside the protected area^8^.

Identification using RS techniques is based on the differences in the spectral reflectance of the objects, which are registered in the raster images. The spectral reflectance depends on many factors, including the species composition^9^. Therefore, it is possible to identify vegetation using RS. The significant number of Natura 2000 natural habitats can be effectively mapped with the use of RS data and machine learning (ML) methods, which was proven by research conducted in recent years^6,7^. Due to limiting factors, the spectral reflectance for different types of vegetation may be similar^10^. Furthermore, natural and semi-natural vegetation are complex areas, and classification using RS techniques can be difficult and does not always meet the expected results^11^. Particular types of vegetation occurring in natural areas are usually heterogeneous, and individual units can be similar in species composition and physiognomy. Natura 2000 habitats and areas are characterised by different ∝-diversity and β-diversity, and these factors can influence the accuracy of habitat mapping^12^. Additionally, the same habitat located in different areas may differ in these features, which makes it difficult to develop a universal identification method^13^. The diversity is the result of their floristic distinctiveness from other habitats. The Natura 2000 habitats with one characteristically dominant species (low ∝-diversity, e.g., heaths, predominantly *Calluna vulgaris*) have a unique spectral signature and are therefore likely to classify with high accuracy using RS^14^. The situation is different with meadows, which can be formed by numerous different species, including morphologically very diverse grasses (high β-diversity). For example, fresh meadows might be populated by *Arrhenatherum elatius*, *Poa pratensis,* and *Festuca rubra* and thus may be more difficult to classify^15^. The spectral resolution and the spectral range of used images must enable the correct identification of the object^16^. Multispectral images have up to a dozen wide bands, whereas hyperspectral data are characterised by hundreds of narrow bands, which makes it easier to differentiate objects. This shows that the level of accuracy acquired in the classification of individual habitats is based on the resolution of the hyperspectral and multispectral images^17^.

Mapping using machine learning may result in insufficient accuracy, and the accuracy affects the practical use of habitat mapping. The accuracy of the acquired map is verified using statistical parameters such as producer accuracy (PA) related to omission error, user accuracy (UA) related to commission error, and accuracy for each class (F1-score). It is impossible to determine acceptable and sufficient accuracy; the highest accuracy is sought in research.

One of the key elements in classification, including Natura 2000 habitats mapping, is choosing the optimal dataset^18,19^. It was performed using data obtained from the satellite level^6,20^ and aerial level^15,21^. The most frequently used were satellite data (especially free-of-charge Sentinel-2 or Landsat 8 images), with aerial data being used less often. Multispectral images are mainly acquired on the satellite level, whereas hyperspectral are on the airborne level, but nowadays high-resolution satellite sensors are also available.

The classifications performed in different studies resulted in very different accuracies, from F1 = 0 for habitat code 6230^21^ to F1 = 0.95 for mires 7140^19^. It is worth mentioning that better results are obtained by using a multi-time image series compared to one date, so it can be stated that using multitemporal images improves the accuracy of classification^15^. Among the classifiers, one of the most effective was the Random Forest (RF) machine learning algorithm^19^.

However, no proven comparisons are done under experimental conditions of the classification of Natura 2000 habitats using the hyperspectral and multispectral data. According to the literature review, the difference in the accuracy of the Natura 2000 habitat classification based on two types of data cannot be stated. The research was conducted in various areas using different reference datasets. As a result, it is impossible to compare the quality of the hyperspectral and multispectral data, or to compare different case studies. For example, heaths were classified using hyperspectral data in five different publications, resulting in an F1 accuracy ranging from 0.28^21^ to 0.90^22^. Based on three different publications, where multispectral data were used, the F1 accuracy varied from 0.44^23^ to 0.93^24^. Considering the accuracies of habitat identification acquired in different studies, it is impossible to determine which data is better and the differences between hyperspectral and multispectral images.

Existing maps, acquired using conventional methods, are used in the management of protected areas. Their flaws include not only the lack of verification, but also the way they are acquired, mainly by field measurements. Such maps are obtained using traditional methods and are rarely compared with machine learning results based on RS. At the same time, such a comparison is not easy due to different techniques of acquisition^25,26^. Machine learning results using remote sensing images are more detailed than conventional mapping^27^.

Therefore, within this analysis, three research questions were formulated:

1. What is the difference in the classification accuracy of individual Natura 2000 habitats using hyperspectral and multispectral images based on HySpex and Sentinel-2 images?
2. Do maps acquired with the use of ML provide knowledge useful for the management of protected areas?
3. Is the utility in nature conservation the same for maps acquired using HS and S2 data?

In this analysis, the HySpex hyperspectral images and Sentinel-2 images were used to identify Natura 2000 habitats. Differences in classification accuracy were determined for: heaths (Natura 2000 code—4030), meadows (1340, 6410, 6440, 6510), grasslands (6230) and mires (7140). To identify the factors influencing the accuracy of classification, the HS data were resampled to a spatial resolution of 10 m which resulted in two different datasets: S2 10 m and HS 10 m. For the HS data, Minimum Noise Fraction (MNF) and Spectral Indices (SI) were used for classification using ML techniques—the RF algorithm. For the S2 data, multitemporal datasets from one vegetation season (spectral bands, MNF, and SI) were tested. Moreover, an additional aim of the analysis was to map the Natura 2000 habitat distribution using acquired RF models for HS and S2 data. The maps have been compared with Natura 2000 habitat maps from the conservation plan in terms of utility.

## Data and methods

### Study area and object of the study

**Figure 1:**
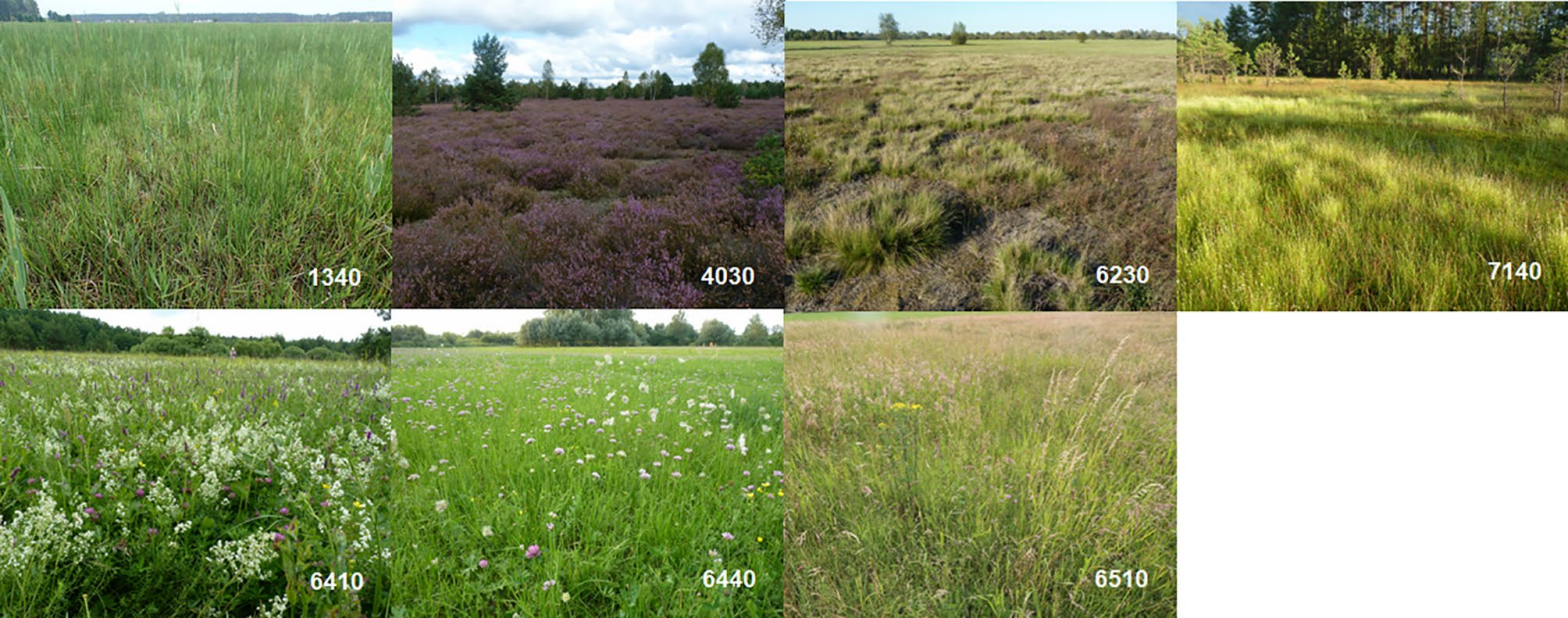
The images of analysed Natura 2000 habitats. The full names of the habitats are given in Table 1. Figure created by the authors using Inkscape (https://inkscape.org). Pictures of the habitats were taken by the authors of the article.

Surveys were performed for seven Natura 2000 habitats (Table 1, Fig. 1). These are the most common non-forest Natura 2000 habitats in Poland's agricultural space. They differ from each other in several features, but they also show similarities^17^. The selected habitats represent 4 different ecosystems: meadows, grasslands, heaths, and mires. Analysed habitats are characterised by different ∝-diversity and β-diversity, as shown (Table 1) descriptively on the basis of the species composition of references polygons and literature data. According to Whitaker’s idea ∝-diversity refers to internal floristic richness within a habitat patch, β-diversity describes the floristic richness between patches^28,29^. General description of the Natura 2000 habitats can be found in the Interpretation Manual of European Union Habitats—EUR28^30^. Two of the surveyed habitats are among the priority ones. Detailed information on the analysed habitats, including their floristic diversity within and between the patches, is described in publications of Natura 2000 habitats in Poland^31–34^.

**Table 1:** Analysed Natura 2000 habitats.

Natura 2000 codes	Habitat type	Type of ecosystem	∝-diversity	β-diversity
1340^a^	Inland salt meadows	Meadows	High	High
4030	European dry heaths	Heaths	Low	Low
6230^a^	Species-rich *Nardus grasslands*, on siliceous substrates in mountain areas (and submountain areas, in Continental Europe	Grasslands	NA1—low KR1—high	NA1—low KR1—high
6410	*Molinia meadows* on calcareous, peaty, or clayey, silt-laden soils (*Molinion caeruleae*)	Meadows	High	High
6440	Alluvial meadows of river valleys of the *Cnidion dubii*	Meadows	High	Low
6510	Lowland hay meadows (*Alopecurus pratensis*, *Sanguisorba officinalis*)	Meadows	High	High
7140	Transition mires and quaking bogs	Mires	Low	High

^a^Priority Natura 2000 habitat.

**Figure 2:**
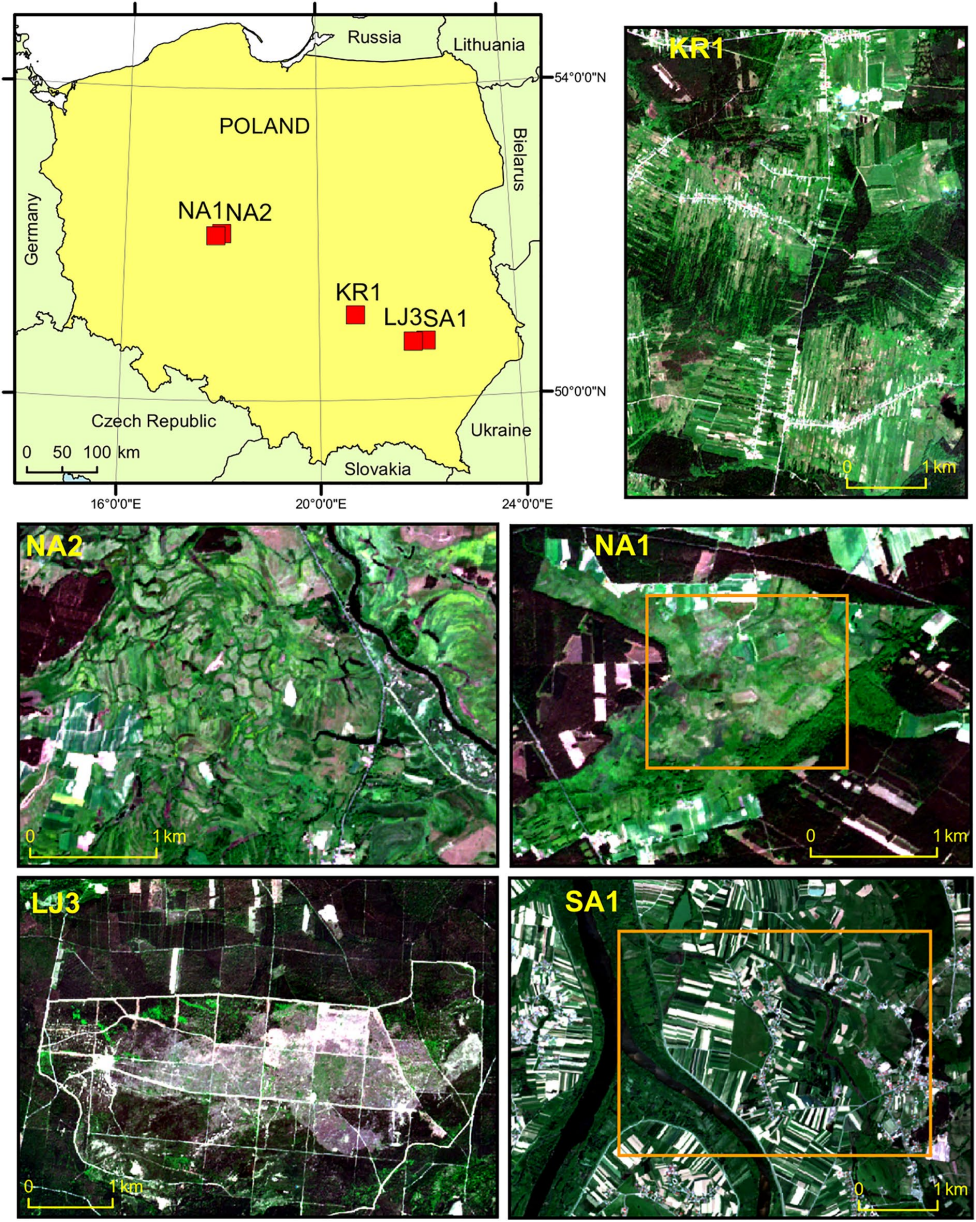
Study sites: KR1—PLH260001 Dolina Krasnej and PLH260012 Uroczysko Pięty; NA1 and NA2—PLH300009 Ostoja Nadwarciańska; LJ3—PLH060031 Uroczyska Lasów Janowskich; SA1—PLH180020 Dolina Dolnego Sanu. Orange squares highlight the areas presented as maps in the results section, background images are Sentinel-2 RGB compositions. Figure created by the authors using ArcMap 10.6.1 software (https://support.esri.com/en/Products/Desktop/arcgis-desktop/arcmap).

The research was carried out in five study sites located within five Special Areas of Conservation (SAC): Ostoja Nadwarciańska (area code PLH300009), Dolina Krasnej (area code PLH260001), Uroczysko Pięty (area code PLH260012), Uroczyska Lasów Janowskich (area code PLH060031) and Dolina Dolnego Sanu (area code PLH180020) (Fig. 2, Table 2).

**Table 2:** The number and date of acquired polygons in 2017.

Area	Data acquisition	Number of polygons acquired during field measurements								
		1340	4030	6230	6410	6440	6510	7140	Background	Sum
KR1	08.06–14.06		10	13	53				189	265
LJ3	05.06–07.06		34					32	142	208
NA1	23.05–28.05	19			27				166	212
NA2	20.05–25.05			17		50			154	221
SA1	05.06–07.06					22	13		114	149
Sum		19	44	30	80	72	13	32	765	1055

Ostoja Nadwarciańska (area code PLH300009) is one of the best-preserved landscapes of a typical lowland river. Two study sites have been established in this location: NA1 (10 km^2^) and NA2 (17 km^2^). Site NA1 has a complex of meadows and rushes surrounded by forests, where habitats with codes 1340 and 6410 occur together: 1340 on local depressions and 6410 on local elevations. Despite the significant floristic distinctiveness of the studied habitats, some of the patches have indicator species of both habitats. The relief of NA2 area is quite varied and rich in fluvial forms. The non-forest communities are predominantly rushes, meadows, and pastures, as well as dry grasslands. Habitats 6440 and 6230 are not adjacent to each other; moreover, they are characterised by a lack of similarity in physiognomy and species composition.

The next research area covers two Special Areas of Conservation: Dolina Krasnej (area code PLH260001) and Uroczysko Pięty (PLH260012). It has been denoted as KR1 (44 km^2^) and is located in the highland. It is partially located in a natural depression between two ranges of hills, partially in the valley of the Krasna River and its tributaries. The area is significantly diversified in terms of geomorphological, soil, and moisture conditions, which results in an extremely wide variety of vegetation. There are very diverse types of plant communities, both forest and non-forest. In this area, three Natura 2000 habitats with codes 4030, 6230, and 6410 were analysed. Patches of habitats 4030 and 6230 usually adjoin each other, forming mosaic and transitional forms. In many cases, this results in a clear similarity of species composition and physiognomy. On the contrary, the patches of habitat 6410 are partially found in separate locations, and sometimes they are adjacent to habitat 6230 with common indicator species.

The research site labelled as LJ3 is located in the eastern part of Uroczyska Lasów Janowskich Special Area of Conservation (area code PLH060031) and covers an area of 41 km^2^. The LJ3 study site is in a lowland area. This is a sandy plain, varied with dune hills and areas with no outflow, dominated by less diverse forest communities. The non-forest area is dominated by moors and patches of dry grasslands. A small area is occupied by semi-natural communities, meadows, and pastures. There is also a complex of peat bogs in the eastern part of the area. Two Natura 2000 habitats were analysed in the LJ3 study site: 4030 and 7140. Habitat 7140 was characterised by species composition and physiognomy distinct from other habitats. Moreover, it was present only in the eastern part of the study area, where patches of habitat 4030 were not recorded. Patches of habitats 4030 occurred only in the western part of the area and were directly adjacent to each other.

The Dolina Dolnego Sanu Special Area of Conservation (area code PLH180020), designated the SA1 study site, is located in the valley of the Lower San River at the estuary section to the Vistula River. The terrain is typical of large rivers and includes elements such as the valley floor, terraces, and oxbow lakes. The agricultural landscape dominates on the surface and is a mosaic of meadows, pastures, and farmlands. Forest communities are less common. The Natura 2000 habitats 6440 and 6510 were analysed in this area. Most of the patches occur in other locations and are not adjacent to each other. Some similarities can be found when comparing the physiognomy and species composition of both habitats.

### On-ground reference data

Synchronous with the acquisition of aerial hyperspectral data, on-ground reference data were collected on the distribution of individual Natura 2000 habitats and other vegetation types and land cover forms. A total of 1055 ground reference polygons were collected, 290 for habitats and 765 for background (Table 2). In each reference polygon, information about the habitat code and plant composition was recorded. The number of polygons distributed within the classes is related to the proportion between the classes in each study area.

The reference polygons were squares with side 10 m. A Sentinel-2 data spatial grid was used to locate the S2 pixels, and the reference square was located in this grid. The square centre coordinates were recorded in the field using a GNSS MobileMapper 120 (with real-time differential correction), obtaining a measurement accuracy of 0.2–0.5 m. The polygons were set up so that they were distributed as evenly as possible within the study area and at the same time were representative of the variability of the analysed habitats and area.

### Maps of management plan

For two examined Natura 2000 sites—Dolina Dolnego Sanu (PLH180020) and Ostoja Nadwarciańska (PLH300009)—current and official maps presenting the distribution of Natura 2000 habitats were available. The maps were provided by the Regional Directorates for Environmental Protection and were made independently as part of ongoing inventories or work on their conservation plan. The maps were obtained using traditional field mapping. It should be assumed that these maps present an average level of detail and accuracy in determining the range of Natura 2000 habitats at the disposal of state level authorities managing the individual areas. For these two sites, a comparative analysis of the maps was acquired using HySpex, Sentinel-2 images and Management Plan (MP) (Fig. 2). In this way, classification methods were compared with traditional field mapping.

### Airborne and satellite data

For sites studied in May and June 2017, hyperspectral images were acquired with 1 m special resolution using two HySpex scanners: VNIR-1800 and SWIR-384 (Table 3). The details of the overflights and radiometric, geometric, and atmospheric correction can be found in previous studies^17,35^. The time in vegetation season was chosen based on previous analyzes and literature^18^.

**Table 3:** The number and date of acquired images in 2017.

Area	[km^2^]	No. of images	Type of data	Acquisition date (number of the day in month)					
				May	June	July	Aug	Sep	Oct
KR1	44	10	S2	18, 28	–	–	08, 11, 16, 31	27, 30	2, 17
		1	HS	–	1	–	–	–	–
LJ3	41	7	S2	–	1		3, 30	9, 29	2, 17
		1	HS	–	2	–	–	–	–
NA1	35	9	S2	15	1, 21	–	3, 30	9, 29	2, 17
		1	HS	–	2	–	–	–	–
NA2	10	7	S2	18, 28	–	30	11	28, 30	18
		1	HS	19	–	–	–	–	–
SA1	17	8	S2	18, 28	20	30	11	28, 30	19
		1	HS	19	–	–	–	–	–

Multispectral S2 images were acquired from vegetation season 2017 (May–November) (Table 3) using Sentinel Data Hub. Multitemporal images were used due to higher accuracy possible to achieve compared to single image analysis^15,24^. Images for three different granules were used: T33UXT (for NA1 and NA2 areas; eight scenes), T34UDB (LJ3 and SA1 areas; thirteen scenes), and T34UDB (KR1 area; ten scenes). All scenes have cloud cover of less than 90%. Some of the images were acquired as surface reflectance (2Ap processing level), and the rest were reflectance on the top of the atmosphere (1C level). In this case, it was necessary to perform atmospheric correction, which was done using the Sen2Cor module in ESA SNAP software. As a result, 31 Sentinel-2 images were chosen for further analysis (Table 3).

### Raster data processing

For the classification, two different datasets were prepared: multitemporal S2 images and HS airborne data. The data was in 10 m resolutions to analyse how spectral resolution influences the classification accuracy regardless of spatial resolution. For both sensors, similar types of data were compared. These datasets were chosen based on previous studies conducted on Natura 2000 habitats identification^15,19^ and data dimensionality reduction for classification^36,37^.

Each band of Sentinel-2 scenes were resampled in ESA SNAP software to 10 m spatial resolution using the nearest neighbour kernel. The S2 bands were combined in multispectral images and imported into the ENVI 5.3 software^38^. All scenes were resized to the extent of five research areas (NA1, NA2, KR1, LJ3, and SA1) based on the extent of the HS data. No clouds were noticed except in the scene for KR1 on 28/05/2017—cumulus clouds were above the forests. This type of land cover was masked during the analysis, to ensure each image was processed the same as others. To produce multitemporal S2 datasets, all images (spectral bands and SI) for each area were stacked, resulting in a multiband image, from 77 bands for NA1 and LJ3 to 110 spectral bands for KR1. To remove noise and add other information, MNF transformation was performed the same as for HS images. The 30 MNF bands were chosen for each study site based on the eigenvalues. With the use of the Spectral Indices tool in ENVI 5.3, 37 SIs were calculated for the multitemporal S2 datasets^39^.

To analyse data in the same spatial resolution, the HS data were resized to fit S2 pixels: 1 m HS images were resampled to 10 m spatial resolution using the S2 pixels’ extent—S2 spatial grid. The resampling was performed in ENVI 5.3 software using Resize Data tool, where the size of the output pixel was defined as 10 m. The images were calculated using nearest neighbour resampling. For the 10 m HS images, MNF transformation was performed and 30 first bands were chosen based on the eigenvalues, visual analysis, and previous studies^15,19^. Next, 65 indices (SI) were calculated in ENVI 5.3 using the Spectral Indices tool^39^.

### Natura 2000 habitats classification

**Figure 3:**
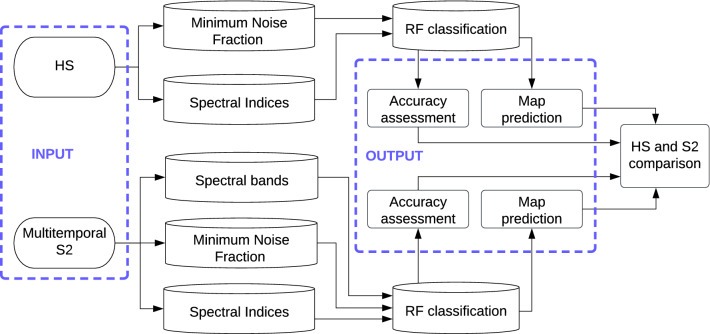
The procedure comparing HS and S2 data. Figure created by the authors using Inkscape (https://inkscape.org).

The classification was performed on both datasets using a RF classifier^40^. An iterative approach was used to divide the reference dataset into training and validation sets, fit the model, assess its accuracy, and acquire the maps. The procedure for acquiring the result was based on the average F1 value, because by using this parameter each class was equally important regardless of the class coverage. For HS images, 30 MNF bands and SI were combined. For multitemporal S2 spectral bands, 30 MNF bands and SI were stacked, and classification was performed on each study area for two different datasets: HS and S2 (Fig. 3).

The classification was performed using the Scikit-learn Python library RF classifier^41^. Although all the processing was designed on a pixel basis, the reference dataset was prepared in the vector format of polygons. They were divided into 50% training and 50% validation sets using the stratified random draw method (used e.g. train_test_split from Scikit-learn). The polygons were rasterised (used gdal) and processed on a pixel basis. The RF classifier was applied to a training dataset using the following parameters: the number of trees in RF: 100; the minimum number of samples per split: 2; the minimum number of samples at leaf node: (1) The trained model was then applied to all the pixels contained by the validation polygons (used gdal). The confusion matrix was built from the reference and predicted classes of all validation pixels (used Scikit-learn). From the confusion matrix, the accuracy measures were calculated: Overall Accuracy (OA) and F1 score for each class. The process of random sampling, rasterization, model fitting, prediction and accuracy assessment was iterated fifty times.

### Statistical analysis

The objective of the first part of the statistical analysis was to define the effectiveness of each Natura 2000 habitat classification depending on the image spectral resolution. The analysis was dedicated to answering the first research question: What is the difference in the classification accuracy of individual Natura 2000 habitats using hyperspectral and multispectral images based on HS and S2 images? The average F1 values for each Natura 2000 habitat on the five study sites were analysed by comparing their distribution from fifty classification iterations for the S2 and HS data. The calculations were performed in Statistica 14.0.0.15^42^. It was checked whether the mean F1 values for HS were greater than those for S2. The non-parametric Mann–Whitney U test was used to analyse whether the differences were statistically significant. The non-parametric test was used because not all F1 values datasets for areas have a normal distribution.

### Maps

**Figure 4:**
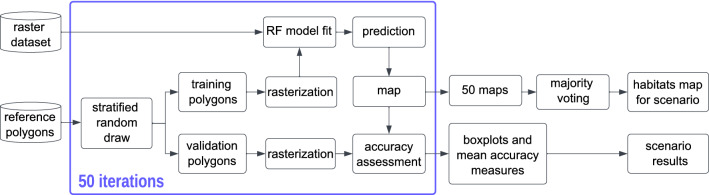
Map production process. Figure created by the authors using Inkscape (https://inkscape.org).

For both datasets, the habitat prediction map was generated. To produce the map of all fifty, fitted models were predicted on the whole study site (Fig. 4.). Then fifty maps were stacked, and the final class was assigned to each pixel by the majority voting.

**Figure 5:**
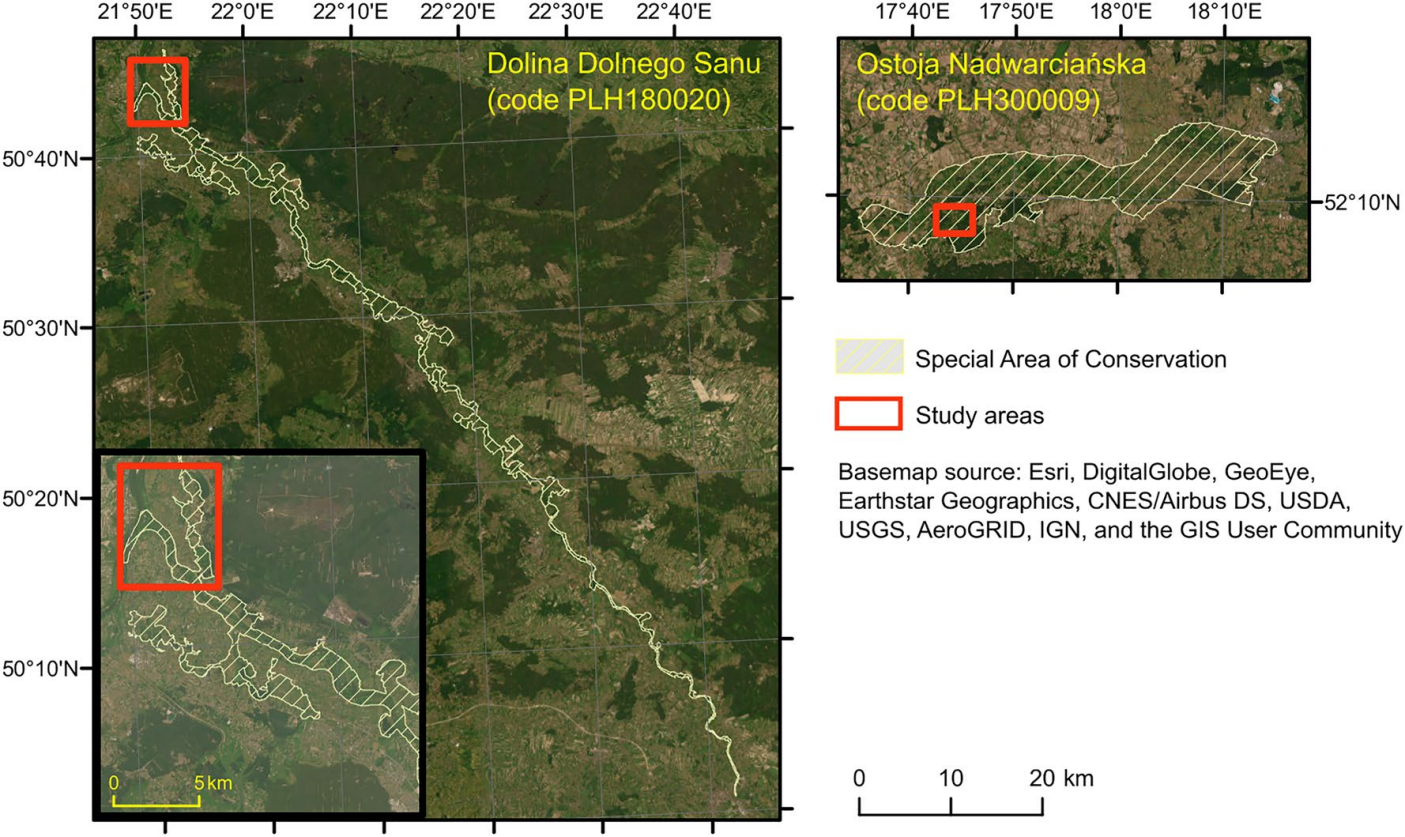
The region covered by Special Areas of Conservation Natura 2000 area, and study areas NA1 and SA1 chosen for visualisation. Figure created by the authors using ArcMap 10.6.1 software (https://support.esri.com/en/Products/Desktop/arcgis-desktop/arcmap).

The obtained classification results were compared with the range of Natura 2000 habitats indicated in the Management Plan of the two areas NA1 and SA1. It should be mentioned that the study areas analysed in this study cover only part of the Special Areas of Conservation (Fig. 5).

For each map, the number of patches and the area occupied by Natura 2000 habitat was determined: 1340 and 6410 in the NA1 area and 6440 and 6510 in SA1. Difference maps were then made for the MP-HS, MP-S2 and HS-S2 combinations. These difference maps have been generalised by removing polygons smaller than two pixels. The Sankey flow diagrams were then developed to show changes between habitat areas on three types of data. The diagrams show changes in the area for pixels that have been indicated as a Natura 2000 habitat on at least one of the maps. The Sankey diagram is a data visualisation technique dating from 1898^43^ commonly used in physics and engineering to display energy flow and have a limited presence in remote sensing literature.

## Results

### The differences in the classification accuracy of individual Natura 2000 habitats using hyperspectral and multispectral images

**Figure 6:**
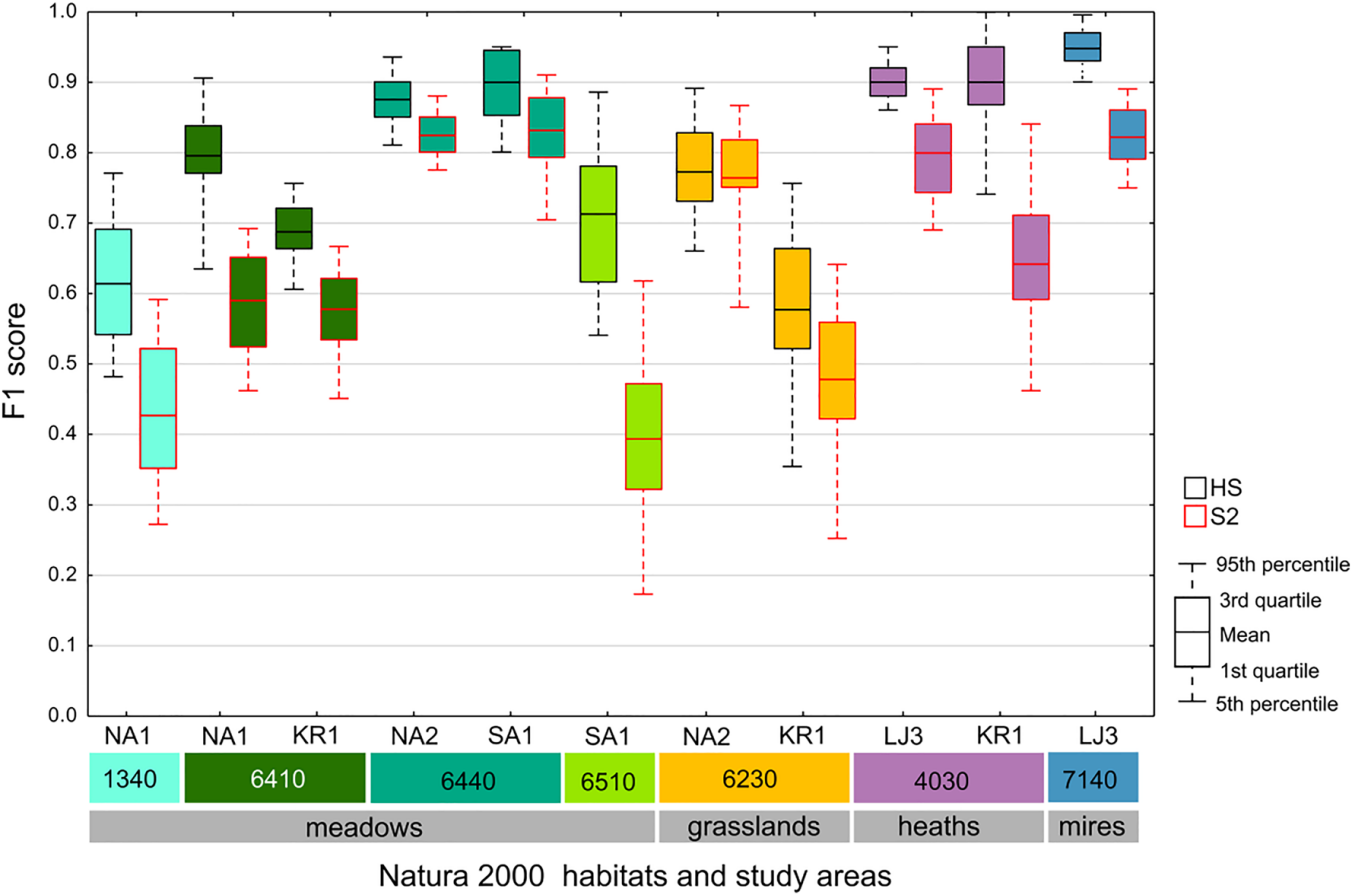
The distribution of F1 accuracy values for each Natura 2000 habitat. The F1 value was acquired from confusion matrices calculated based on fifty cal/val polygons stratified random sampling (Table 2). Figure created by the authors using RAWGraphs 2.0 beta (https://app.rawgraphs.io/).

A comparative analysis of the accuracy of individual Natura 2000 habitats indicates that the classification accuracy differs between HS and S2. The accuracy based on HS data is always higher than S2 data—the average difference is around 0.14. In most cases, the difference is statistically significant (Fig. 6). The F1 score acquired for Natura 2000 habitats for each area was higher for HS data compared to S2 images, but the value of the differences is not constant—the highest difference (0.32) was noticed for habitat 6510 in area SA1. The differences were statistically significant except for habitat 6230 in the NA2 area, which was also the smallest (0.009). It should also be noted that, in almost every case, the PA was lower compared to the UA, so the habitat classes are rather underestimated than overestimated (see Supplementary Material).

The highest values of F1 accuracy for HS were obtained for mires (code 7140) on area LJ3 (F1 = 0.95). A slightly lower F1 score was obtained for both areas of heaths (code 4030, F1 = 0.90) and alluvial meadows (code 6440, F1 between 0.87 and 0.90). The lowest accuracy was acquired for grassland (code 6230) on the KR1 area (F1 = 0.57) and salt meadows on NA1 (code 1340, F1 = 0.61). The most stable results F1 values calculated for 50 iterations were obtained in the case of mires (code 7140), where the F1 values varied from 0.89 to 1. The biggest difference in F1 values occurred in the case of grasslands (code 6230) in the KR1 area.

For four habitats that were analysed in two different areas, it was possible to compare the accuracy between the study sites and check the stability of the results. Similar F1 values were noticed in the case of heaths (code 4030), and alluvial meadows (code 6440); the difference in mean F1 accuracy between the two areas was less than 0.03. The results for grasslands (code 6230) were the least stable; the difference in the average accuracy of F1 between KR1 and NA2 was equal to 0.20.

In the case of S2 data, the highest accuracy was achieved in four cases: alluvial meadows (code 6440) for both areas (F1 = 0.82 for NA2 and F1 = 0.83 for SA1), mires (code 7140, F1 = 0.82), and in one of the heathland areas (code 4030, F1 = 0.80). The lowest accuracies were noticed for meadows 6510 (F1 = 0.39) and 1340 (F1 = 0.42). For lowland hay meadows (code 6510) the F1 values are also very diverse. High accuracy stability within habitats mapped on two areas was achieved for two types of meadows: alluvial meadows (code 6440), where the difference of F1 was less than 0.01, and *Molinia* meadows (code 6410), where the difference was just over 0.01. In the case of heathlands (4030) and grasslands (6230), the results should be considered unstable; the differences in F1 between areas were equal to 0.16 and 0.29, respectively.

Generally, results for HS were better than for S2, however there were noticeable differences in the decrease in the F1 score between S2 and HS. Regardless of the area, a relatively small decrease in accuracy was noticed for alluvial meadows (code 6440). The average decrease in F1 value was equal to 0.07 for both areas. The highest F1 decrease (0.32) was noticed for lowland hay meadows (code 6510). Also, for this habitat, a substantial difference in producer accuracies was found (0.63 for HS and 0.27 for S2) (see Supplementary Material). It can be stated that this habitat is underestimated based on S2 images. Diverse results dependent on the study area were achieved for grasslands (code 6230). For NA2 the difference was very small (0.01) and statistically not significant. For KR1 the difference was equal to 0.1—*Molinia* meadows (code 6440) and heaths (code 4030). For these three habitats, at least in one area, the decrease of F1 accuracy was around 0.1, which may indicate the possibility of achieving similar classification results using both sensors. However, the results strongly depend on the study area.

### Comparison of Natura 2000 habitat maps created by HS and S2 data classification with maps from Management Plans

A comparison of the Natura 2000 habitat distribution maps resulting from the classification of HS and S2 data, with those derived from MP, shows very large differences between them in both the NA1 and SA1 areas. These discrepancies concern both the number of patches, their size, and their total area.

**Figure 7:**
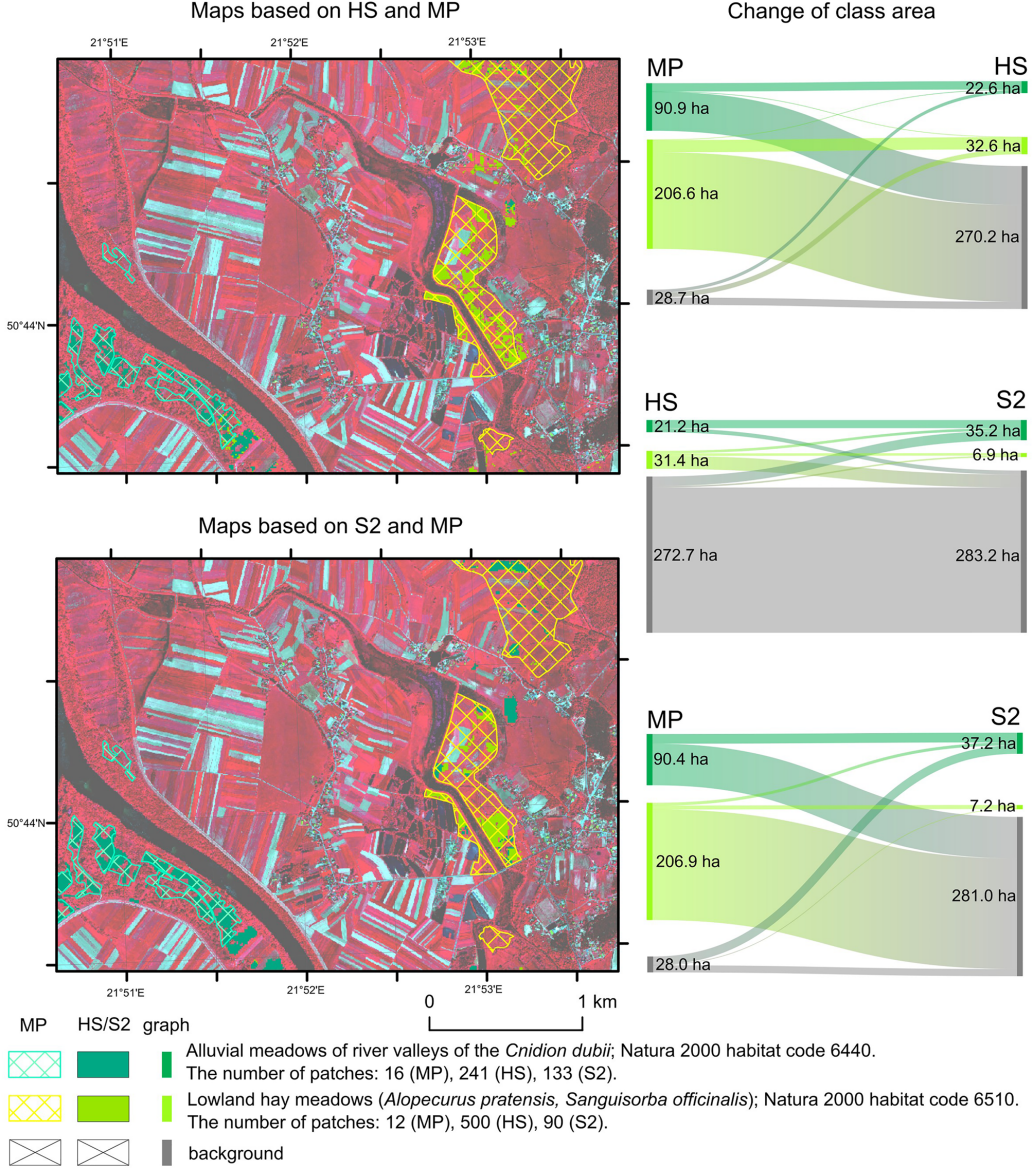
The classification results in the SA1 area based on HS and S2 images compared with MP maps. On the left are presented Sankey graphs showing the are difference for each map. The aerial photos used in the map background are CIR-composition quick look taken from HySpex data acquired for this study as part of the HabitARS project. Figure created by the authors using ArcMap 10.6.1 software (https://support.esri.com/en/Products/Desktop/arcgis-desktop/arcmap) and SankeyMATIC (https://sankeymatic.com).

Within the SA1 site of the 90.6 hectares of 6440 habitat patches indicated in the MP, only 15.80 ha were confirmed as a result of HS data classification, and 17.10 ha from S2 data (Fig. 7). Additionally, 6.76 ha of new patches were shown on the HS data and 20.11 ha on the S2 data. Even greater differences between the maps can be seen when analysing the distribution of habitat 6510. Of the 207.0 hectares of habitat shown in MP, the HS classification results confirmed only 22.95 hectares and indicated 9.61 hectares of new habitat patches.

In the case of S2 classification results, only 6.50 hectares were confirmed, and 0.62 hectares of new habitat patches were previously unregistered. The concordance of the MP maps with HS, calculated as the percentage of confirmed habitat area with MP on the HS map, can therefore be estimated at 13.06%, while the MP maps against S2 are estimated at 7.93%. The significant difference between the MP, S2 and HS maps also relates to the number of patches and their fragmentation, e.g., the extent of habitat 6440 in the maps from the MP was mapped as 16 patches while 241 patches were identified in the HS maps and 133 patches in the S2 data.

The differences between the maps created by classifying HS and S2 data are large and apply to both 6510 and 6440 habitats. As a result of the classification of S2 data, a significantly larger total area of habitat 6440 is visible (an increase mainly at the expense of background patches). The opposite trend was observed for habitat 6510. A significant part of the areas classified on the HS data was assigned to the background class on the S2 data. The concordance of the distribution map of the two studied habitats resulting from the S2 data classification against the HS map can be assessed as average. In the case of habitat 6440, 14.20 ha is the area jointly shown as habitat on HS and S2 data to the total area of patches on HS data = 21.3 ha and S2 = 35.3 ha.

**Figure 8:**
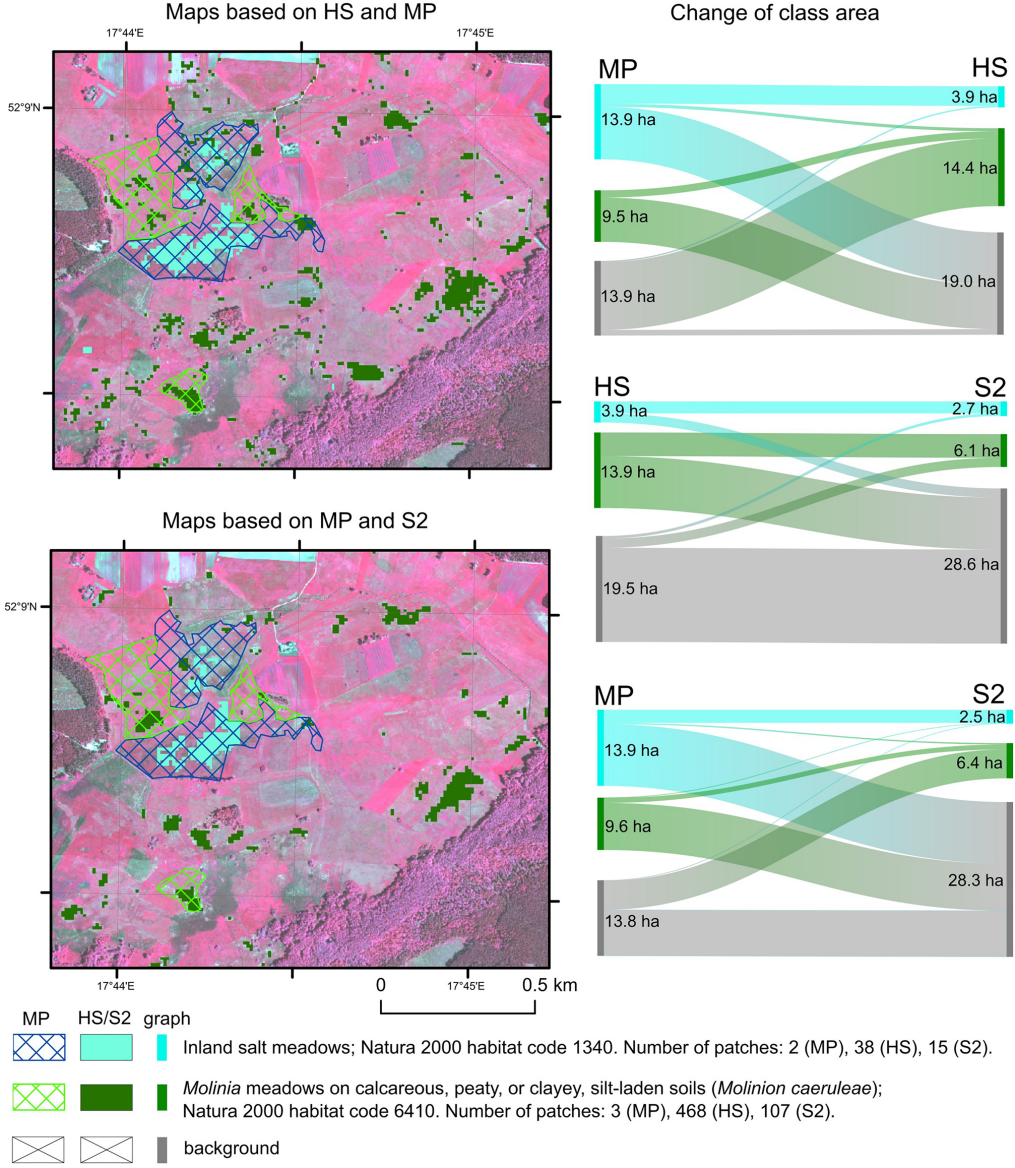
The classification results in the NA1 area based on HS and S2 images compared with MP maps. On the left are presented Sankey graphs showing the area difference for each map. The aerial photos used in the map background are CIR-composition quick look taken from HySpex data acquired for this study as part of the HabitARS project. Figure created by the authors using ArcMap 10.6.1 software (https://support.esri.com/en/Products/Desktop/arcgis-desktop/arcmap) and SankeyMATIC (https://sankeymatic.com).

In the case of the NA1 area, of the 13.92 ha of 1340 habitat patches identified according to the MP, only 3.62 ha were confirmed using HS data and 2.38 ha using S2 data (Fig. 8). In addition, new habitat patches were found using classification; 0.27 ha using HS data classification and 0.16 ha with S2 data. The differences between the distribution maps of habitat 1340 on the MP data and the classification results can be considered very large. According to the mentioned calculation, the classification results indicate a significantly smaller area of habitat 1340 in the inventoried area.

For habitat 6410, the differences in the maps are even greater. Of the 9.53 ha of habitat patches present on the MP maps, only 1.33 ha were confirmed using the HS data and 0.90 using the S2 data. At the same time, classification of HS data indicates the presence of 13.11 ha of habitat patches (HS data classification) and 5.54 ha for S2 results in places outside the habitat range on the MP data. The concordance of the MP maps with HS, calculated as a percentage of confirmed habitat area with MP on the HS map, can thus be estimated at 21.11%, while the MP maps against S2 are estimated at 13.98%.

The differences between the MP maps and those produced by the classification process relate to the size of the patches. As in the case of the SA1 area, the results of HS and S2 are characterised by much greater fragmentation of the patches. The concordance between the two classification results should also be assessed as average for both habitats. For habitat 1340, 2.18 hectares is the area jointly indicated as surveyed habitat on the HS and S2 data, while for habitat 6410, 4.35 hectares is the area of habitat indication which is consistent with both classification results.

## Discussion

### The differences in mapping accuracy between hyperspectral and multispectral data

No studies so far have been conducted comparing the accuracy of the identification of different types of Natura 2000 non-forest habitats based on hyperspectral and multispectral data. Classification accuracy comparisons have only been possible by comparing the results of different studies (Table 4). Based on this it is not possible to conclude that one data type is more useful for habitat mapping than the other. The main reason for this is the lack of comparability of results (i.e., the inability to eliminate the influence of factors other than spectral resolution). Individual publications differ not only in terms of the datasets but also, for example, in the processing used, the date of data acquisition, the diversity of areas, and the amount of reference data. In our earlier research, it was proven that HS data have better differentiation capabilities compared to S2 data^17^.

**Table 4:** The overview of the accuracies for individual Natura 2000 habitats acquired in this study and from the literature review. The table includes only the results of studies using hyperspectral data (Hyper) or multispectral data (Multi) and the sources of accuracies for classes of Natura 2000 habitats.

Code Natura 2000	RS data	F1 score from this study	F1 score from literature	Source
Meadows (1340)	Hyper	0.61	No data	No data
	Multi	0.42	No data	No data
Meadows (6410)	Hyper	0.69; 0.79	0.73	^44^
	Multi	0.58; 0.59	0.85	^45^
			0.74–0.85	^46^
			0.74	^47^
Meadows (6440)	Hyper	0.87; 0.90	0.83	^20^
	Multi	0.82; 0.83	No data	No data
Meadows (6510)	Hyper	0.71	0.72	^48^
			0.85	^18^
	Multi	0.39	0.89	^49^
			0.77–0.90	^46^
Grassland (6230)	Hyper	0.57; 0.77	0.92	^22^
			0.00	^21^
	Multi	0.48; 0.76	0.73	^46^
			0.74	^24^
Heaths (4030)	Hyper	0.90; 0.90	0.70	^50^
			0.40	^21^
	Multi	0.64; 0.80	0.94–0.97	^51^
Mires (7140)	Hyper	0.95	0.80	^22^
			0.63	^44^
			0.92–0.95	^19^
	Multi	0.82	0.85	^45^
			0.71	^24^
			0.34	^47^

The problem of comparing the utility of hyperspectral and multispectral images to vegetation mapping was analysed in terms of classification of other vegetation classes, such as plant communities. For example, the accuracies for HS and S2 were comparable when classifying plant communities in the Tatra Mountains; F1 varied from 0.76 to 0.90 depending on the dataset^52^. Similar accuracies for hyperspectral and multispectral data (0.90 and 0.93 respectively) were acquired in habitat mapping in parts of North West England^53^. On the other hand, the classification OA was higher (0.78) for the hyperspectral CASI data compared to the S2 (OA = 0.69 in the classification of natural vegetated coastal areas on the Pakri Islands^54^. This implies that classification at a finer spectral resolution can improve vegetation classification, which was also proven by other studies^10,55^. Based on performed analysis and results of statistical tests it can be stated that the accuracy based on HS data is always higher than for S2 data, but the difference is not constant—the average difference was equal to 0.14. The difference between the F1 accuracy varies depending on the habitat and area.

In this study of three types of meadows—salt meadows (1340), *Molinia* meadows (6410) and lowland hay meadows (6510)—the F1 accuracy was higher for HS data than for S2 images. Generally, accuracies were from 0.61 to 0.79 for HS and from 0.39 to 0.59 in the case of S2 (Fig. 6). Therefore, using hyperspectral data could be essential for recognising the habitat. The biggest differences (above 0.32) were noticed for lowland hay meadows 6510. These habitats are characterised by very high ∝-diversity and β-diversity which requires using high spectral resolution^18,46^. Individual patches of 6410 habitat have high β-diversity and may be dominated by different species, such as *Molinia caerulea, Betonica officinalis, Selinum carvifolia, Succisa pratensis or Serratula tinctoria*. Generally, meadows are characterised by the dominance of grasses and very high species richness, which can cause difficulty in remote sensing analysis^10^. The species composition in Natura 2000 habitats can be similar to the neighbouring plant communities, but can also differ, for example, by only a few species. Therefore, hyperspectral data should be used to identify these meadow habitats.

A relatively small decrease in accuracy between using HS and S2 data was noticed for alluvial meadows (code 6440); the average decrease in F1 value was equal to 0.07 for both areas (Fig. 6). Also, average accuracies for both sensors are quite high, with values above 0.82. Good results can be related to the fact that the habitat is characterised by low ∝-diversity and β-diversity with the dominance of one species on each site—*Cnidium dubium* on NA1 and *Allium angulusum* on SA1. These are characteristic of this habitat and do not occur in the background class. The relatively small difference in accuracy between HS and S2 data is probably due to the fact that using multitemporal S2 images makes it possible to utilise the phenological changes in alluvial meadows. In the previously conducted research on meadow habitats, it was proved that the use of multitemporal images increases the accuracy of the results by about 6%^24^, so very low spectral resolution of S2 in relation to HS can be significantly compensated by the use of multitemporal data. In the case of this habitat, using multitemporal S2 images is sufficient for the correct mapping.

Quite different results were noticed for *Naruds stricta* grasslands (6230), where in the NA1 area the difference in accuracies for HS and S2 is irrelevant (0.009) and the accuracies were around 0.76 (Fig. 6). In the KR1 area, the differences in accuracies for HS and S2 are bigger, but still less than 0.1. Small differences can be related to low ∝-diversity and β-diversity. In this case, multispectral images are sufficient for identification.

Good results were noticed in the case of heaths, which has low ∝-diversity and β-diversity. The results for the LJ3 area showed that the differences in F1 between HS and S2 were around 0.1 and the accuracies were high—0.90 for HS and 0.80 for S2 (Fig. 6). On the other hand, accuracy was dependent on the study area: the differences in accuracy between HS and S2 for KR1 were very high—above 0.26. In the case of this habitat, the results for HS images are repeatable, so by using HS images it is possible to properly identify habitats. These results are different compared to the literature^21,50,51^. The differences between KR1 and LJ3 areas can be explained by differences in reference data—a smaller number of polygons were used on KR1, and classes with a smaller number of reference polygons can produce underestimated classes, which were proven in the previous study^56^. Also, the lower accuracy on KR1 can be caused by the low occurrence of species *Calluna vulgaris* in the background class.

Similar results were noticed for mires habitat (code 7140); the accuracies were quite high (above 0.82 for HS and S2 data) and the differences slightly exceeded 0.1 (Fig. 6), but the results were acquired in one study area. Similar accuracies and their differences for hyperspectral and multispectral images were achieved earlier—the accuracies for hyperspectral are higher compared to multispectral, but the differences are not high (Table 4). The relatively good results can be explained by the low ∝-diversity individual patches. This habitat is possible to map using images with both spectral resolutions, but results for hyperspectral data are slightly better compared to multispectral images.

In general, it can be stated that the accuracies for HS compared to S2 are higher. Based on the research, it can be concluded that hyperspectral data are obligatory for meadows 1340, 6410, and 6510, recommended for heaths 4030 and mires 7140, and unnecessary in the case of meadows 6440 and grasslands 6230. Unfortunately, the acquisition of airborne hyperspectral data is not free unlike satellite data, which limits the utility of data. Satellite hyperspectral data may be a solution to the problem. Two satellite hyperspectral sensors such as PRISMA and HyMap are now available^57,58^. Both sensors have 30-m spatial resolution, around 250 bands, and cover spectrum from visible to shortwave infrared. Also, simulated EnMAP data were previously successfully used for monitoring vegetation communities^59^. In parallel, it is necessary to develop multitemporal analyses.

### Applicability of obtained results

The use of remote sensing to monitor protected Natura 2000 habitats is widely described^6,7^. The use of remote sensing for mapping valuable habitats was already analysed at the beginning of the twenty-first century^25,27^. In this case, the greater detail of maps acquired using RS techniques in relation to traditional maps was emphasised, despite having poorer sensors and simpler algorithms than ML. The analyses conducted in this study for two Special Areas of Conservation (PLH300009 Ostoja Nadwarciańska and PLH180020 Dolina Dolny Sanu) indicate very large differences between the maps of habitat distribution acquired using RS techniques and conventional maps used by regional nature protection services (Figs. 7, 8). Maps created using RS provide new knowledge concerning the protected habitats distribution. As it has been proven, using ML and both the HS and S2 images allowed for the identification of new patches of Natura 2000 habitats (Figs. 7, 8) and more accurate delineation of the border of previously determined patches. It also did not confirm the existence of the previously shown patches. It can therefore be concluded that the use of RS provides new knowledge about the distribution of habitats, which also allows better monitoring of habitats outside protected areas; a common problem today^8^. The lack of, or limited, knowledge of the distribution of valuable habitats is still one of the main concerns of EU countries^60^. Remote sensing provides solutions that can help solve this problem on a regional or national scale.

However, the accuracy assessment of the hyperspectral and multispectral data classification indicated underestimation and overestimation of the habitat area (see Supplementary Material). The relatively large differences in accuracy between hyperspectral and multispectral images affect their utility. Although both types of data provide new knowledge valuable in the context of monitoring, the mapping results based on the S2 images were characterised by a significantly lower PA value compared to HS or its lower stability between areas (see Supplementary Material). Therefore, it should be assumed that the S2 data are characterised by the lower utility for Natura 2000 habitats monitoring because it is not possible to identify a significant part of the patches. This is particularly true for habitats 1340, 6410 and 6510. For some of the habitats (6440), no significant difference can be observed in the habitat maps made using HS and S2. Based on obtained results, it can be stated that the monitoring of Natura 2000 habitats should be developed based on RS data, in particular using hyperspectral images, which are more universal. This is because it was possible to acquire satisfactory results for all the studied habitats with RS data, while S2 data only showed results for some of the habitats.

## Conclusions

Based on conducted classifications using hyperspectral and Sentinel-2 data in five Natura 2000 sites and seven Natura 2000 habitats, it can be concluded that:

1. The accuracy of Natura 2000 habitat classification using HS data is always higher than using S2 data. On average, the difference in F1 accuracy for habitat classes is 0.14.
2. The differences in accuracy between hyperspectral and multispectral data are high for habitats characterised by very high ∝-diversity and β-diversity (like lowland hay meadows 6510) and low for habitats with low ∝-diversity and β-diversity (like heaths 4030).
3. Using S2 multitemporal images it is possible to identify meadows 6440 and grassland 6230. For heaths 4030 and mires 7140, using HS data improved the results, but it is also possible to acquire general distribution of these classes, whereas HS images are obligatory for mapping meadows 1340, 6410 and 6510.

Based on a comparison of the maps produced by the classification of hyperspectral and Sentinel-2 data for the two protected areas, with the traditional maps produced for the conservation plans, it can be concluded that:

1. The HS and S2 data make it possible to create maps that provide a great deal of new knowledge about the distribution of Natura 2000 habitats, which is necessary for the management of protected areas.
2. Maps created using HS provide, for most of the studied habitats, data of higher utility for management in conservation. The main difference between HS and S2 maps is a significantly higher omission error for S2 data.
3. Remote sensing today provides the tools to effectively monitor the distribution of non-forest Natura 2000 habitats.

## Supplementary Information

Supplementary Table 1.

## Data Availability

The datasets generated during and/or analysed during the current study are available from the corresponding author on reasonable request.

## Abbreviations

RS: Remote sensing S2 Sentinel-2
HS: HySpex
MNF: Minimum noise fraction
SI: Spectral indices
OA: Overall accuracy
ML: Machine learning
MP: Management plan
UA: User accuracy
RF: Random Forest
PA: Producer accuracy
