# Supplementary material for: The utility of airborne hyperspectral and satellite multispectral images in identifying Natura 2000 non-forest habitats for conservation purposes
Source: Sci Rep. 2023 Mar 20;13:4549. doi: 10.1038/s41598-023-31705-6 (PMC10027895; doi:10.1038/s41598-023-31705-6)
Supplement: Supplementary file 1 — Supplementary Table 1. [file 41598_2023_31705_MOESM1_ESM.pdf]

**The utility of airborne hyperspectral and satellite multispectral images in identifying Natura 2000 non-forest habitats for conservation purposes**

Anna Jarocińska, Dominik Kopeć, Jan Niedzielko, Justyna Wylazłowska, Anna Halladin-Dąbrowska, Jakub Charyton, Agnieszka Piernik, Dariusz Kamiński

**Supplementary Information**

Table 1. The average of Producer (PA) and User Accuracy (UA) calculated for HySpex (HS) and Sentinel-2 (S2) data based on 50 iterations.

| Area | Class | PA | | UA | |
|---|---|---|---|---|---|
| | | HS | S2 | HS | S2 |
| NA1 | 1340 | 0.46 | 0.29 | 0.96 | 0.85 |
| | 6410 | 0.72 | 0.46 | 0.90 | 0.84 |
| | background | 0.98 | 0.98 | 0.90 | 0.85 |
| NA2 | 6230 | 0.74 | 0.71 | 0.81 | 0.84 |
| | 6440 | 0.84 | 0.78 | 0.92 | 0.88 |
| | background | 0.96 | 0.95 | 0.92 | 0.90 |
| LJ3 | 4030 | 0.85 | 0.71 | 0.96 | 0.92 |
| | 7140 | 0.95 | 0.78 | 0.94 | 0.87 |
| | background | 0.98 | 0.96 | 0.96 | 0.89 |
| SA1 | 6440 | 0.85 | 0.78 | 0.96 | 0.90 |
| | 6510 | 0.63 | 0.27 | 0.85 | 0.80 |
| | background | 0.98 | 0.98 | 0.94 | 0.89 |
| KR1 | 4030 | 0.88 | 0.52 | 0.94 | 0.88 |
| | 6230 | 0.50 | 0.38 | 0.72 | 0.70 |
| | 6410 | 0.56 | 0.46 | 0.90 | 0.77 |
| | background | 0.96 | 0.95 | 0.85 | 0.81 |
